# Author Correction: Tidal wetland resilience to sea level rise increases their carbon sequestration capacity in United States

**DOI:** 10.1038/s41467-019-13800-3

**Published:** 2019-12-11

**Authors:** Faming Wang, Xiaoliang Lu, Christian J. Sanders, Jianwu Tang

**Affiliations:** 1 Xiaoliang Research Station for Tropical Coastal Ecosystems and Key Laboratory of Vegetation Restoration and Management of Degraded Ecosystems, South China Botanical Garden, Chinese Academy of Sciences, Guangzhou, 510650 P.R. China; 2 Center of Plant Ecology, Core Botanical Gardens, Chinese Academy of Sciences, Guangzhou, 510650 P.R. China; 3 Southern Marine Science and Engineering Guangdong Laboratory (Guangzhou), Guangzhou, 511458 P.R. China; 4 State Key Laboratory of Estuarine and Coastal Research and Institute of Eco-Chongming, East China Normal University, Shanghai, 201100 P. R. China; 5 The Ecosystems Center, Marine Biological Laboratory, Woods Hole, MA 02543 USA; 6 State Key Laboratory of Soil Erosion and Dryland Farming on the Loess Plateau, Institute of Soil and Water Conservation, Northwest A&F University, Yangling, Shaanxi China 712100; 7 National Marine Science Centre, School of Environment, Science and Engineering, Southern Cross University, Coffs Harbour, NSW 2450 Australia

**Keywords:** Climate-change ecology, Wetlands ecology, Carbon cycle, Biogeochemistry

Correction to: *Nature Communications* 10.1038/s41467-019-13294-z, published online 28 November 2019.

The original version of this Article contained an error in the spelling of the author Faming Wang, which was incorrectly given as Farming Wang. This has now been corrected in both the PDF and HTML versions of the Article.

