# Supplementary material for: Tidal wetland resilience to sea level rise increases their carbon sequestration capacity in United States
Source: Nat Commun. 2019 Nov 28;10:5434. doi: 10.1038/s41467-019-13294-z (PMC6883032; doi:10.1038/s41467-019-13294-z)
Supplement: Supplementary file 2 — Supplementary Information [file 41467_2019_13294_MOESM2_ESM.pdf]

Title: Tidal wetland resilience to sea level rise increases their carbon sequestration capacity in United States

Farming Wang et al.

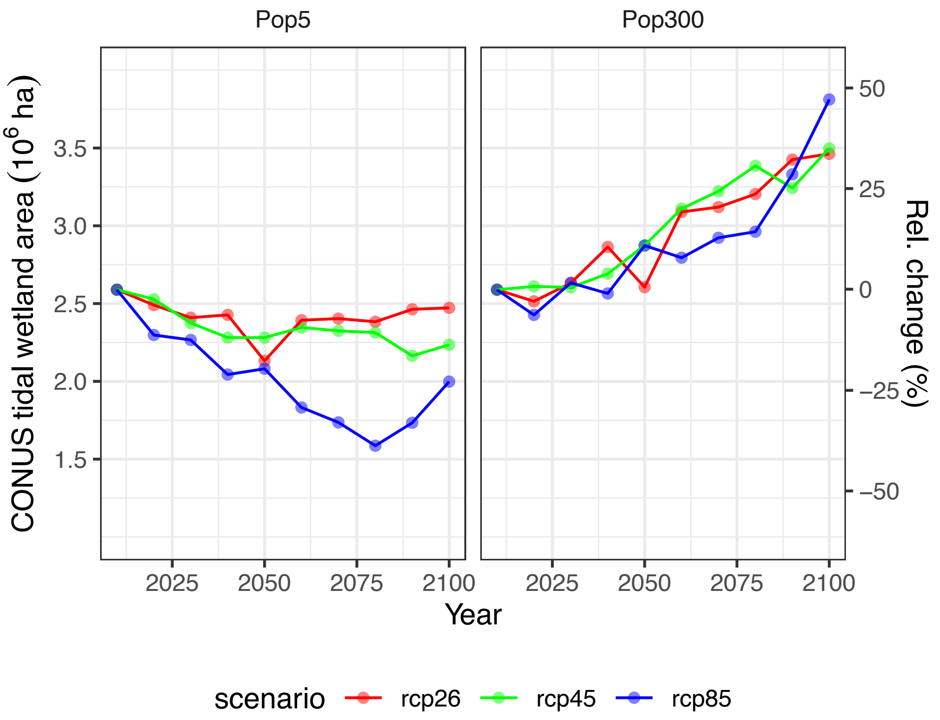

Supplementary Figure 1. The modeled CONUS tidal wetland area changes by Schuerch, et al. [1] under different RCP scenarios and Population density thresholds.

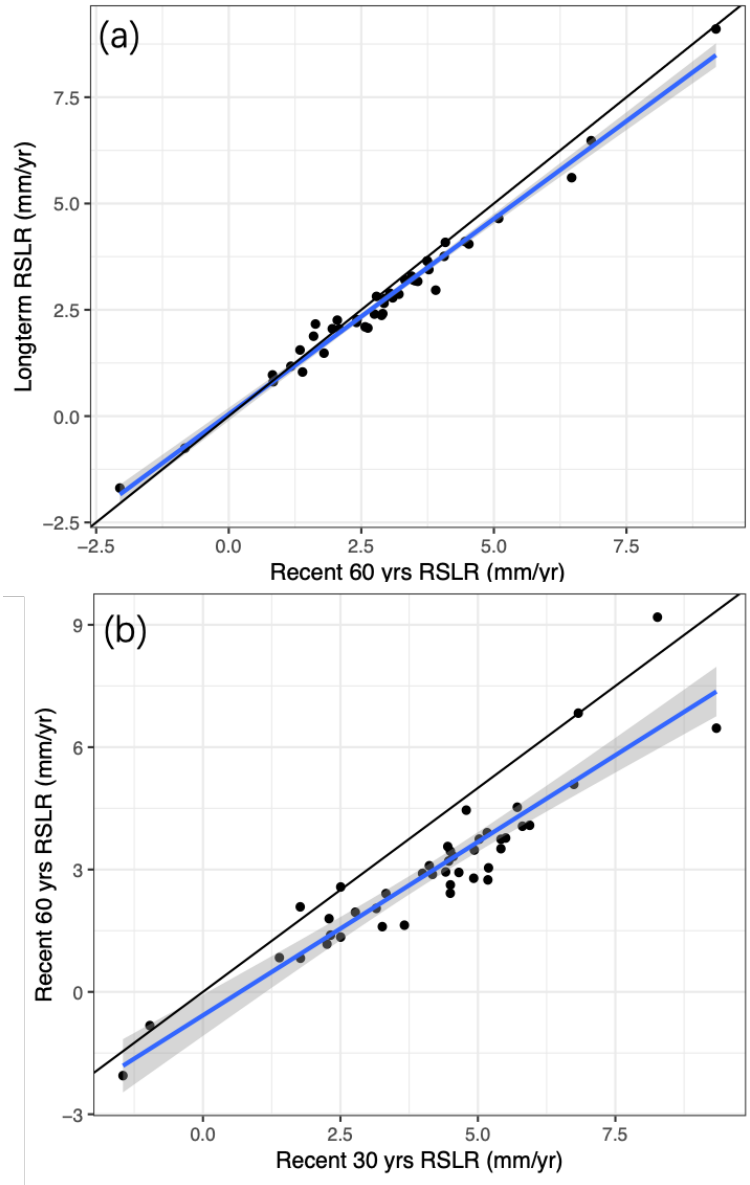

Supplementary Figure 2. The relationships among different time scale RSLR rates recorded by tidal gauges (continuous records over 60 yrs) along the conterminous U.S. (CONUS) coastal lines (n=43). (a) The relationship between recent 60 yrs RSLR and full time range long-term RSLR (over 60 yrs) and (b) the relationship between recent 60 yrs RSLR and recent 30 yrs RSLR rate. The linear relationship ($y=0.85025x-0.57453$, $R^2=0.85$ and $P<0.001$) was used to standardize the other 29 CONUS sites where the records ranged between 30 yrs and 60 yrs. The solid black lines represent the line of $y=x$.

Supplementary References

1 Schuerch, M. *et al.* Future response of global coastal wetlands to sea-level rise. *Nature* **561**, 231-234, doi:10.1038/s41586-018-0476-5 (2018).

2 Anisfeld, S. C., Tobin, M. & Benoit, G. Sedimentation rates in flow-restricted and restored salt marshes in Long Island Sound. *Estuaries* **22**, 231-244, doi:10.2307/1352980 (1999).

3 Armentano, T. V. & Woodwell, G. M. Sedimentation rates in a Long Island marsh determined by ^{210}Pb dating. *Limnol. Oceanogr.* **20**, 452-456, doi:10.4319/lo.1975.20.3.0452 (1975).

4 Boyd, B. M. & Sommerfield, C. K. Marsh accretion and sediment accumulation in a managed tidal wetland complex of Delaware Bay. *Ecol. Eng.* **92**, 37-46, doi:10.1016/j.ecoleng.2016.03.045 (2016).

5 Breithaupt, J. L., Smoak, J. M., Smith, T. J. & Sanders, C. J. Temporal variability of carbon and nutrient burial, sediment accretion, and mass accumulation over the past century in a carbonate platform mangrove forest of the Florida Everglades. *Journal of Geophysical Research: Biogeosciences* **119**, 2032-2048, doi:10.1002/2014jg002715 (2014).

6 Bricker-Urso, S., Nixon, S. W., Cochran, J. K., Hirschberg, D. J. & Hunt, C. Accretion rates and sediment accumulation in Rhode Island salt marshes. *Estuaries* **12**, 300-317, doi:10.2307/1351908 (1989).

7 Cahoon, D. R. Recent Accretion in Two Managed Marsh Impoundments in Coastal Louisiana. *Ecol. Appl.* **4**, 166-176, doi:10.2307/1942126 (1994).

8 Cahoon, D. R. & Lynch, J. C. Vertical accretion and shallow subsidence in a mangrove forest of southwestern Florida, U.S.A. *Mangroves Salt Marshes* **1**, 173-186, doi:10.1023/a:1009904816246 (1997).

9 Callaway, J. C., Borgnis, E. L., Turner, R. E. & Milan, C. S. Carbon Sequestration and Sediment Accretion in San Francisco Bay Tidal Wetlands. *Estuaries and Coasts* **35**, 1163-1181, doi:10.1007/s12237-012-9508-9 (2012).

10 Callaway, J. C., DeLaune, R. D. & Patrick, W. H. Sediment accretion rates from four coastal wetlands along the Gulf of Mexico. *J. Coast. Res.* **13**, 181-191 (1997).

11 Campana, M. L. *The effect of Phragmites australis invasion on community processes in a tidal freshwater marsh.* M.S. Thesis, thesis, College of William and Mary, (1998).

12 Carey, J. C., Moran, S. B., Kelly, R. P., Kolker, A. S. & Fulweiler, R. W. The Declining Role of Organic Matter in New England Salt Marshes. *Estuaries and Coasts*, 1-14, doi:10.1007/s12237-015-9971-1 (2015).

13 Church, T. M. *et al.* Marsh sediments as records of sedimentation, eutrophication and metal pollution in the urban Delaware Estuary. *Mar. Chem.* **102**, 72-95, doi:10.1016/j.marchem.2005.10.026 (2006).

14 Church, T. M., Lord, C. J. & Somayajulu, B. L. K. Uranium, thorium and lead nuclides in a Delaware salt marsh sediment. *Estuar. Coast. Shelf Sci.* **13**, 267-275, doi:10.1016/S0302-3524(81)80025-4 (1981).

15 Clark, J. S. & Patterson, W. A. Pollen, PB-210, and Opaque Spherules: An Integrated Approach to Dating and Sedimentation in the Intertidal Environment. *Journal of Sedimentary Petrology*

54, 1251-1265 (1984).
16 Corman, S. S., Roman, C. T., King, J. W. & Appleby, P. G. Salt Marsh Mosquito-Control Ditches: Sedimentation, Landscape Change, and Restoration Implications. *J. Coast. Res.* **283**, 874-880, doi:10.2112/jcoastres-d-11-00012.1 (2012).
17 Craft, C. Freshwater input structures soil properties, vertical accretion, and nutrient accumulation of Georgia and U.S. tidal marshes. *Limnol. Oceanogr.* **52**, 1220-1230 (2007).
18 Craft, C. B. Tidal freshwater forest accretion does not keep pace with sea level rise. *Global Change Biol.* **18**, 3615-3623, doi:10.1111/gcb.12009 (2012).
19 Craft, C. B., Seneca, E. D. & Broome, S. W. Vertical Accretion in Microtidal Regularly and Irregularly Flooded Estuarine Marshes. *Estuar. Coast. Shelf Sci.* **37**, 371-386, doi:10.1006/ecss.1993.1062 (1993).
20 Delaune, R. D., Patrick, W. H. & Buresh, R. J. Sedimentation rates determined by 137Cs dating in a rapidly accreting salt marsh. *Nature* **275**, 532-533 (1978).
21 Delaune, R. D., Smith, C. J. & Patrick, W. H. Sedimentation patterns in a gulf coast backbarrier marsh: Response to increasing submergence. *Earth Surface Processes and Landforms* **11**, 485-490, doi:10.1002/esp.3290110503 (1986).
22 Donnelly, J. P. & Bertness, M. D. Rapid shoreward encroachment of salt marsh cordgrass in response to accelerated sea-level rise. *Proc. Natl. Acad. Sci. U.S.A* **98**, 14218-14223, doi:10.1073/pnas.251209298 (2001).
23 Drake, K., Halifax, H., Adamowicz, S. C. & Craft, C. Carbon Sequestration in Tidal Salt Marshes of the Northeast United States. *Environment Management* **56**, 998-1008, doi:10.1007/s00267-015-0568-z (2015).
24 Drexler, J. Z. *et al.* A Long-Term Comparison of Carbon Sequestration Rates in Impounded and Naturally Tidal Freshwater Marshes Along the Lower Waccamaw River, South Carolina. *Wetlands* **33**, 965-974, doi:10.1007/s13157-013-0456-3 (2013).
25 Goldberg, E. D., Griffin, J. J., Hodge, V., Koide, M. & Windom, H. Pollution history of the Savannah River Estuary. *Environ. Sci. Technol.* **13**, 588-594, doi:10.1021/es60153a012 (1979).
26 Gonneea, M. E., O'Keefe Suttles, J. A. & Kroeger, K. D. (U.S. Geological Survey data release, 2018).
27 Goodman, J. E., Wood, M. E. & Gehrels, W. R. A 17-yr record of sediment accretion in the salt marshes of Maine (USA). *Mar. Geol.* **242**, 109-121, doi:10.1016/j.margeo.2006.09.017 (2007).
28 Greiner, M. & Hershner, C. Analysis of wetland total phosphorus retention and watershed structure. *Wetlands* **18**, 142-149, doi:10.1007/bf03161451 (1998).
29 Hatton, R. S., Delaune, R. D. & Patrick, W. H. Sedimentation, accretion, and subsidence in marshes of Bararia Basin, Louisiana. *Limnol. Oceanogr.* **28**, 494-502 (1983).
30 Kastler, J. A. & Wiberg, P. L. Sedimentation and Boundary Changes of Virginia Salt Marshes. *Estuar. Coast. Shelf Sci.* **42**, 683-700, doi:10.1006/ecss.1996.0044 (1996).
31 Kearney, M. S. & Stevenson, J. C. Island Land Loss and Marsh Vertical Accretion Rate Evidence for Historical Sea-Level Changes in Chesapeake Bay. *J. Coast. Res.* **7**, 403-415 (1991).
32 Kearney, M. S. & Ward, L. G. Accretion rates in brackish marshes of a Chesapeake Bay estuarine tributary. *Geo-Mar. Lett.* **6**, 41-49, doi:10.1007/bf02311695 (1986).
33 Khan, H. & Brush, G. S. Nutrient and metal accumulation in a fresh-water tidal marsh. *Estuaries* **17**, 345-360, doi:10.2307/1352668 (1994).
34 Kim, G., Alleman, L. Y. & Church, T. M. Accumulation records of radionuclides and trace metals

in two contrasting Delaware salt marshes. *Mar. Chem.* **87**, 87-96, doi:10.1016/j.marchem.2004.02.002 (2004).

35 Loomis, M. J. & Craft, C. B. Carbon Sequestration and Nutrient (Nitrogen, Phosphorus) Accumulation in River-Dominated Tidal Marshes, Georgia, USA. *Soil Sci. Soc. Am. J.* **74**, doi:10.2136/sssaj2009.0171 (2010).

36 Lynch, J. C., Meriwether, J. R., McKee, B. A., Vera-Herrera, F. & Twilley, R. R. Recent Accretion in Mangrove Ecosystems Based on 137 Cs and 210 Pb. *Estuaries* **12**, 284-299, doi:10.2307/1351907 (1989).

37 Marchio, D. A., Savarese, M., Bovard, B. & Mitsch, W. J. Carbon Sequestration and Sedimentation in Mangrove Swamps Influenced by Hydrogeomorphic Conditions and Urbanization in Southwest Florida. *Forests* **7**, doi:10.3390/f7060116 (2016).

38 McCaffrey, R. J. & Thomson, J. in *Advances in Geophysics* Vol. Volume 22 (ed Saltzman Barry) 165-236 (Elsevier, 1980).

39 Muzyka, L. J. *Pb^{210} chronology in a core from the Flax Pond marsh, Long Island* Master thesis, SUNY-Stony Brook, (1976).

40 Neubauer, S. C., Anderson, I. C., Constantine, J. A. & Kuehl, S. A. Sediment Deposition and Accretion in a Mid-Atlantic (U.S.A.) Tidal Freshwater Marsh. *Estuar. Coast. Shelf Sci.* **54**, 713-727, doi:10.1006/ecss.2001.0854 (2002).

41 Noe, G. B., Hupp, C. R., Bernhardt, C. E. & Krauss, K. W. Contemporary Deposition and Long-Term Accumulation of Sediment and Nutrients by Tidal Freshwater Forested Wetlands Impacted by Sea Level Rise. *Estuaries and Coasts* **39**, 1006-1019, doi:10.1007/s12237-016-0066-4 (2016).

42 Nyman, J. A. Organic Matter Fluxes and Marsh Stability in a Rapidly Submerging Estuarine Marsh. *Estuaries and Coasts* **18**, 207-218, doi:10.2307/1352631 (1995).

43 Nyman, J. A., deLaune, R. D. & Patrick Jr, W. H. Wetland soil formation in the rapidly subsiding Mississippi river deltaic plain: Mineral and organic matter relationships. *Estuar. Coast. Shelf Sci.* **31**, 57-69 (1990).

44 Nyman, J. A., Walters, R. J., Delaune, R. D. & Patrick, W. H. Marsh vertical accretion via vegetative growth. *Estuarine Coastal and Shelf Science* **69**, 370-380, doi:10.1016/j.ecss.2006.05.041 (2006).

45 Orson, R. A. & Howes, B. L. Salt Marsh development studies at Waquoit Bay, Massachusetts: Influence of geomorphology on long-term plant community structure. *Estuar. Coast. Shelf Sci.* **35**, 453-471, doi:10.1016/S0272-7714(05)80025-3 (1992).

46 Orson, R. A., Simpson, R. L. & Good, R. E. Rates of sediment accumulation in a tidal fresh-water marsh. *Journal of Sedimentary Petrology* **60**, 859-869 (1990).

47 Orson, R. A., Warren, R. S. & Niering, W. A. Interpreting Sea Level Rise and Rates of Vertical Marsh Accretion in a Southern New England Tidal Salt Marsh. *Estuar. Coast. Shelf Sci.* **47**, 419-429, doi:10.1006/ecss.1998.0363 (1998).

48 Parkinson, R. W., Delaune, R. D. & White, J. R. Holocene Sea-Level Rise and the Fate of Mangrove Forests within the Wider Caribbean Region. *J. Coast. Res.* **10**, 1077-1086 (1994).

49 Redfield, A. C. Development of a New England Salt Marsh. *Ecol. Monogr.* **42**, 201-237, doi:10.2307/1942263 (1972).

50 Richard, G. A. Seasonal and environmental variations in sediment accretion in a Long Island salt marsh. *Estuaries* **1**, 29-35, doi:10.2307/1351647 (1978).

51 Semien, J. *Are New England salt marshes keeping up with rising sea levels: comparing sediment accumulation with estimates of net carbon storage based on Eddy flux tower measurements at the Plum Island Estuary?* SES thesis, Dillard University, (2012).
52 Sharma, P., Gardner, L. R., Moore, W. S. & Bollinger, M. S. Sedimentation and bioturbation in a salt marsh as revealed by 210Pb, 137Cs, and 7Be studies12. *Limnol. Oceanogr.* **32**, 313-326, doi:10.4319/lo.1987.32.2.0313 (1987).
53 Smoak, J. M., Breithaupt, J. L., Smith, T. J. & Sanders, C. J. Sediment accretion and organic carbon burial relative to sea-level rise and storm events in two mangrove forests in Everglades National Park. *Catena* **104**, 58-66, doi:10.1016/j.catena.2012.10.009 (2013).
54 Stevenson, J. C., Kearney, M. S. & Pendleton, E. C. Sedimentation and erosion in a Chesapeake Bay brackish marsh system. *Mar. Geol.* **67**, 213-235, doi:10.1016/0025-3227(85)90093-3 (1985).
55 Sturdevant, A., Craft, C. B. & Sacco, J. N. Ecological functions of an impounded marsh and three natural estuarine marshes along Woodbridge River, NY/NJ Harbor. *Urban Ecosystems* **6**, 163-181, doi:10.1023/a:1026356211862 (2002).
56 Thom, R. M. Accretion rates of low intertidal salt marshes in the Pacific Northwest. *Wetlands* **12**, 147-156, doi:10.1007/bf03160603 (1992).
57 Vogel, R. L., Kjerfve, B. & Gardner, L. R. Inorganic Sediment Budget for the North Inlet salt marsh, South Carolina. *Mangoves and Salt Marshes* **1**, 23-35 (1996).
58 Ward, L. G., Kearney, M. S. & Stevenson, J. C. Variations in sedimentary environments and accretionary patterns in estuarine marshes undergoing rapid submergence, Chesapeake Bay. *Mar. Geol.* **151**, 111-134, doi:10.1016/S0025-3227(98)00056-5 (1998).
59 Weis, D. A., Callaway, J. C. & Gersberg, R. M. Vertical accretion rates and heavy metal chronologies in wetland sediments of the Tijuana Estuary. *Estuaries* **24**, 840-850, doi:10.2307/1353175 (2001).
60 Weston, N. B., Neubauer, S. C., Velinsky, D. J. & Vile, M. A. Net ecosystem carbon exchange and the greenhouse gas balance of tidal marshes along an estuarine salinity gradient. *Biogeochemistry* **120**, 163-189, doi:10.1007/s10533-014-9989-7 (2014).
61 Chrzastowski, M. J. *Stratigraphy and geologic history of a Holocene lagoon: Rehoboth Bay and Indian River Bay, Delaware* Ph.D thesis, University of Delaware, (1986).
62 Flessa, K. W., Constantine, K. J. & Cushman, M. K. Sedimentation Rates in a Coastal Marsh Determined from Historical Records. *Chesapeake Science* **18**, 172-176, doi:10.2307/1350858 (1977).
63 Artigas, F. *et al.* Long term carbon storage potential and CO_2 sink strength of a restored salt marsh in New Jersey. *Agricultural and Forest Meteorology* **200**, 313-321, doi:10.1016/j.agrformet.2014.09.012 (2015).
64 Nyman, J. A., Delaune, R. D., Roberts, H. H. & Patrick, W. H. Relationship between vegetation and soil formation in a rapidly submerging coastal marsh. *Mar. Ecol. Prog. Ser.* **96**, 269-279, doi:10.3354/meps096269 (1993).
65 Roman, C. T., Peck, J. A., Allen, J. R., King, J. W. & Appleby, P. G. Accretion of a New England (U.S.A.) Salt Marsh in Response to Inlet Migration, Storms, and Sea-level Rise. *Estuar. Coast. Shelf Sci.* **45**, 717-727, doi:10.1006/ecss.1997.0236 (1997).
