# Supplementary material for: Tidal wetland resilience to sea level rise increases their carbon sequestration capacity in United States
Source: Nat Commun. 2019 Nov 28;10:5434. doi: 10.1038/s41467-019-13294-z (PMC6883032; doi:10.1038/s41467-019-13294-z)
Supplement: Supplementary file 3 — Reporting Summary [file 41467_2019_13294_MOESM3_ESM.pdf]

nature research

Corresponding author(s): Jianwu Tang and Faming Wang

Last updated by author(s): Oct 23, 2019

Reporting Summary

Nature Research wishes to improve the reproducibility of the work that we publish. This form provides structure for consistency and transparency in reporting. For further information on Nature Research policies, see Authors & Referees and the Editorial Policy Checklist.

Statistics

For all statistical analyses, confirm that the following items are present in the figure legend, table legend, main text, or Methods section.

n/a	Confirmed	
☐	☒	The exact sample size (*n*) for each experimental group/condition, given as a discrete number and unit of measurement
☐	☒	A statement on whether measurements were taken from distinct samples or whether the same sample was measured repeatedly
☐	☒	The statistical test(s) used AND whether they are one- or two-sided<br>*Only common tests should be described solely by name; describe more complex techniques in the Methods section.*
☐	☒	A description of all covariates tested
☐	☒	A description of any assumptions or corrections, such as tests of normality and adjustment for multiple comparisons
☐	☒	A full description of the statistical parameters including central tendency (e.g. means) or other basic estimates (e.g. regression coefficient) AND variation (e.g. standard deviation) or associated estimates of uncertainty (e.g. confidence intervals)
☐	☒	For null hypothesis testing, the test statistic (e.g. *F*, *t*, *r*) with confidence intervals, effect sizes, degrees of freedom and *P* value noted<br>*Give P values as exact values whenever suitable.*
☒	☐	For Bayesian analysis, information on the choice of priors and Markov chain Monte Carlo settings
☒	☐	For hierarchical and complex designs, identification of the appropriate level for tests and full reporting of outcomes
☒	☐	Estimates of effect sizes (e.g. Cohen's *d*, Pearson's *r*), indicating how they were calculated

Our web collection on statistics for biologists contains articles on many of the points above.

Software and code

Policy information about availability of computer code

Data collection	A Source Data File, containing the raw data underlying the research and all figures and tables presented in our paper, is available in the Supplementary Information.
Data analysis	The data analysis was conducted by R and SPSS 21.0. The code used in this study is available from F.W. on request.

For manuscripts utilizing custom algorithms or software that are central to the research but not yet described in published literature, software must be made available to editors/reviewers. We strongly encourage code deposition in a community repository (e.g. GitHub). See the Nature Research guidelines for submitting code & software for further information.

Data

Policy information about availability of data

All manuscripts must include a data availability statement. This statement should provide the following information, where applicable:

- Accession codes, unique identifiers, or web links for publicly available datasets
- A list of figures that have associated raw data
- A description of any restrictions on data availability

A Source Data File, containing the raw data underlying the research and all figures and tables presented in our paper, is available in the Supplementary Information. Figure 1 data was extracted from Natioanl Wetland Inventary data (https://www.fws.gov/wetlands/) based on the wetland type code attached in the Supplementary Table S1. The predicted global wetland area change data was extracted from the model by Schuerch et al. 2018 (https://gitlab.com/mark.schuerch/global-coastal-wetland-model.git.). Correspondence and requests for other data and code should be addressed to F.W.

Field-specific reporting

Please select the one below that is the best fit for your research. If you are not sure, read the appropriate sections before making your selection.

☐ Life sciences ☐ Behavioural & social sciences ☒ Ecological, evolutionary & environmental sciences

For a reference copy of the document with all sections, see nature.com/documents/nr-reporting-summary-flat.pdf

Ecological, evolutionary & environmental sciences study design

All studies must disclose on these points even when the disclosure is negative.

Study description	We synthesized C accumulation rate (CAR) in tidal wetlands of the conterminous US, upscaled the CAR to national scale using the National Wetland Inventory dataset, estimated their annual C sequestration and predicted trends based on relative sea level rise (RSLR) scenarios.
Research sample	We compiled 372 data sites, of which 310 sites reported C accumulation rate (CAR), or in which CAR can be calculated based on reported values; and of which 343 sites reported soil accretion rates (SAR) (Table S1).
Sampling strategy	We used the Web of Science (Thomson Reuters, New York, NY) and Google Scholar (Google Inc., Mountain View, CA) to search the literature using the terms: ((accretion)+(tidal wetlands or tidal marsh or mangroves or tidal marsh or coastal wetland or coastal marsh)+(soil or sediments)).
Data collection	Dr. Faming Wang examined 64 published studies (Supplementary Table 1) that reported sediment accretion rates and soil C density or parameters necessary for estimating C density (Bulk density, soil organic matter content, or soil C content) in tidal wetlands of the conterminous US.
Timing and spatial scale	The data sampling collection was till 2018 for the studies in the conterminous US.
Data exclusions	The vertical SAR represented average soil accretion rates from decades to centuries, depending on the different dating methods. The 137Cs and 210Pb dating methods were employed in 317 sites to determine decadal rates of vertical accretion, and 24 sites measured sub-decadal and decadal SAR based on the surface elevation table (SET) methods. Only one study used 14C dating method to rebuild centuries to thousands of years accretion rates 12. The 14C dating method usually has a lower SAR than the method using 137Cs and 210Pb 40. To avoid statistical skew by dating methods, this study was not included in the final CAR calculation.
Reproducibility	The data collection and analysis can be easily repeated based on the methods that we provided.
Randomization	All the collected data was grouped by their vegetation types, and locations (e.g. states, regions and coasts)
Blinding	Not applicable for this kind of data synthesis study.

Did the study involve field work? ☐ Yes ☒ No

Reporting for specific materials, systems and methods

We require information from authors about some types of materials, experimental systems and methods used in many studies. Here, indicate whether each material, system or method listed is relevant to your study. If you are not sure if a list item applies to your research, read the appropriate section before selecting a response.

Materials & experimental systems

n/a	Involved in the study	
☒	☐	Antibodies
☒	☐	Eukaryotic cell lines
☒	☐	Palaeontology
☒	☐	Animals and other organisms
☒	☐	Human research participants
☒	☐	Clinical data

Methods

n/a	Involved in the study	
☒	☐	ChIP-seq
☒	☐	Flow cytometry
☒	☐	MRI-based neuroimaging
